# Is there life beyond the Spanish government’s aid to furloughed employees by COVID-19?

**DOI:** 10.1371/journal.pone.0253331

**Published:** 2021-06-23

**Authors:** Juan Laborda, Pilar Rivera-Torres, Vicente Salas-Fumas, Cristina Suárez

**Affiliations:** 1 Department of Business Administration, Universidad Carlos III, Getafe, Madrid, Spain; 2 Department of Marketing Management and Market Research, Universidad de Zaragoza, Zaragoza, Spain; 3 Department of Business Administration and Organization, Universidad de Zaragoza, Zaragoza, Spain; 4 Department of Economics, Universidad de Alcalá, Alcalá de Henares, Madrid, Spain; The Bucharest University of Economic Studies, ROMANIA

## Abstract

This paper examines the perceptions of firms in April 2020, one month after the Spanish Government declared the state of alarm, about how the COVID-19 pandemic will affect their business activity in the following months, and what employment decisions they expect to make in response. The data for the study was collected by the Government of the region of Aragon (Spain) through a survey of a non-randomly selected sample of firms located in the region. In addition to prospects and intended actions, firms were asked whether or not they had applied for ERTE aid (the Spanish job retention scheme to contain the pandemic crisis). We find that firms participating (voluntarily and anonymously) in the survey anticipated rather well the severity of the effects of the pandemic in the following months. The ERTE aid helped firms to maintain the jobs of their inactive employees, while firms that did not ask for aid responded by laying off employees. Further, the ERTE aid helped to maintain the jobs of furloughed employees, but the firms receiving ERTE aid expected to lay off the same proportion of employees as firms without that aid, controlling for the different anticipated effects of the pandemic in the two groups of firms.

## Introduction

In March 2020, the Spanish government declared a ‘state of alarm’ in order to gain control over the public health problem created by the accelerated spread of the COVID-19 virus. The government decision and later actions (social distancing, confinement…) to turn down the curve of the epidemic had an immediate impact: a severe contraction of economic activity, with a high level of uncertainty about how deep the recession would be and how long it would last. The Spanish government, like most governments across the world [1], took emergency policy decisions to compensate firms for the potential losses from the suspension of non-basic activities, including employment and labor cost subsidies, tax delays, and liquidity provision through loan guarantees.

This paper focuses on one of the public aid programs most commonly adopted across countries: compensating firms for the labor costs of keeping employees temporarily employed, although inactive (i.e. furloughed employees). In Spain, this program was designated ERTE (*Expedientes de Regulación Temporal de Empleo*) and was open to any firm, including the solo self-employed, that could demonstrate a decrease in revenues for reasons directly or indirectly related to the pandemic. In the two weeks period of end of April and early may, the Aragon’s government regional agency IAF (Instituto Aragonés de Fomento) set up a remote, hands-off and anonymous survey to obtain fresh and timely information on how the pandemic was affecting activity and sales prospects of firms in the region, on how firms responded to the pandemic shock, and whether they had asked for ERTE aid or not. The self-selection of the respondents to the survey, with no ex ante assurance of representation, and with firms responding about prospects and intentions, not actual decisions, was the price paid for the timeliness of the data collected, when the official statistics would come only after months of delay. Several other initiatives have been reported on efforts to collect firm data, similar to the survey data collected for this research [2–5].

One year later, much more is known about what has really happened in the region and in the country, but we believe it is still of interest to assess the quality and reliability of the timely but untested data, in two main ways. First, what do prospects and intentions tell us about the reactions of firms in the first weeks of the pandemic; and second, how well does the survey data anticipate what actually happened in the Spanish economy during the following twelve months? Since the collection of this kind of survey data is likely to grow with the facilities provided by the remote collection schemes available in the market, it is necessary to have as much evidence as possible on how well the trade-off between timeliness and opportunity on the one hand, and statistical rigor on the other, really works out. The region of Aragon where the data was collected is representative of the Spanish economy (sometimes also referred as the Spanish Ohio, [6]), and could be used as an interesting case for similar regions within the EU [7], but, again, it is necessary to check on its applicability whenever possible.

With respect to the substantive issues, three research questions will be addressed. The first question of interest for policy makers is if the firms that asked for ERTE aid were indeed those targeted by the policy initiative, namely those with the worst prospects of facing relatively long periods of interrupted activity, and/or worst prospects of revenue losses. This was a necessary condition for the ERTE program serving the goals for which it was created. For policy makers, it was also important to know the relative importance of the observable characteristics of firms, such as size and economic sector, relative to unobservable ones, perceptions about the prospects of inactivity and fall in revenues, in predicting the response to the aid program. Depending on the answers, it would make sense to define the policies of targeted firms in terms of structural characteristics of size and sector, or it would be necessary to rely on private data at the firm level.

The second question of interest is how firms adjust their labor force in response to the anticipation of a fall in demand, both among firms taking the ERTE-aid and among those not taking the aid. The expectation, if the policy action is to be effective, is that firms taking the ERTE aid will adjust most of their labor needs with more- or less-furloughed employees, while those firms with no ERTE aid will adjust their labor needs with layoffs. Therefore, the hypothesis is that the adjustment of employment will be less sensitive to the revenue prospects among firms that are able to use furloughs to adjust labor needs than among firms otherwise.

The third research question is whether the labor aid program did, in fact, save jobs, beyond those saved by furloughing employees who would otherwise have become unemployed. The number of jobs that would have been lost without the labor aid program is unknown, and the survey did not ask firms for the counterfactual (how much jobs would had been lost without the aid program). Within the data limitation, the analysis consists of, first, examining whether the differences in intentions on layoffs of employees between ERTE aid and non-ERTE aid firms are sensitive to controlling for characteristics of firms that may condition their decision to ask for ERTE, or not controlling for them. If the answer is yes then the isolation of the ERTE aid effect in the prospect of layoffs is difficult to identify. As a complementary robustness exercise, the paper proposes a pseudo-counterfactual analysis that consists in comparing, in a difference in difference way, intended layoffs of firms with ERTE aid and predicted layoffs for no ERTE aid firms if the latter had had the same characteristics in terms of prospects of activity and revenues that ERTE aid firms.

With respect to the goal of assessing the representativeness and reliability of the survey-collected data, the paper present two kinds of evidence. First, it compares the distribution of firms that respond to the survey by size classes and sectors, with the distribution of firms that respond to the countrywide survey launched in November of 2020 by the Bank of Spain [8]. Second, it compares the sector and size class averages of intentions on layoffs by firms in Aragon in April 2020, to the respective country level data on actual layoffs reported by the survey of the Bank of Spain, and by the Spanish Statistical Office. Ideally it would have been desirable to ask the same firms that answered the survey what they actually did during the year 2020, in terms of days of activity, declines in revenue, and layoffs, but this has not been possible, among other things because firms answered the survey anonymously.

The results confirm that the COVID-19 pandemic has caused an important and widespread disruption in the activity of Spanish firms. In the aggregate for the whole economy, across economic sectors and across size classes, firms that respond to the survey anticipated quite well what actually happened during the following months of pandemic. Thus, we conjecture that at the individual firm level the intentions in April 2020 anticipated reasonably well what occurred in the following months. Overall, our evidence is encouraging about the utility for policy decisions of the kind of survey data reported in this study. With reasonably reliable data, and with correspondence between intentions and actual decisions, the results indicate that those firms with the least promising prospects for activity and future revenues were more likely to take ERTE-aid, confirming the effectiveness of the program. The sensitivity analysis of employment prospects to revenue perceptions is lower among firms with ERTE-aid, confirming that the furloughing of employees is an alternative to layoffs in response to temporary falls in demand and revenues. The predicted prospects of job losses for the firms that asked for aid—if they had had similar characteristics to those firms that did not—are not statistically different, on average, from the prospects for layoffs of firms not asking for aid. Thus, the ERTE program has only contributed to protect the jobs of the furloughed employees, with no external effects in terms of directly saving jobs of non-furloughed employees.

## Institutional settings and literature review

### Public aid to firms in the pandemic

The public policies set up by the Spanish government to mitigate the socio-economic effects of COVID-19, employment included, are quite similar to those set up by other countries in Europe and around the world. The first policy package was issued on March 12, 2020, and extended later in the same month, as a consequence of the severity of the pandemic (Royal Law-Decrees: 7/2020 of March 12–2020; 8/2020 of March 17; and 9/2020 of March 27). The policy initiatives and aid programs were grouped around labor and liquidity-oriented measures. The former was intended to provide flexibility to firms in responding to the shock, i.e. to continue being operative while, at the same time, preventing the destruction of firms and jobs and, ultimately, avoiding a massive increase in unemployment. The ERTE-aid, together with the subsidies to those solo self-employed who had to suspend their activity, are the most important government aid programs to business firms in Spain. The support to preserve the liquidity of firms had a similar goal, to ensure the continuity in business of firms with a shortage of revenues and liquid assets in the short term, but with an expectation of being solvent in the mid- and long-terms.

All Spanish firms that were forced by COVID-19 to stop or reduce their activity were eligible to request the ERTE-aid program. Firms decided the number of employees who became furloughed, with no limits on eligibility and no minimum period of time contributing to the Social Security System required. The economic subsidy for the firm was 70% of the net salary of furloughed employees, plus a total or partial bonus of contribution to social security paid by the firm. Initially, the program had a duration of three months, but it was later extended until May 31, 2021. Furloughed employees were not allowed to work and those working from home were not eligible for furloughing.

Twenty-three OECD countries already had plans and programs for firms that had to adjust the working time of their employees for major unanticipated reasons, and eight countries introduced new plans and programs in response to COVID-19. German firms, for example, were eligible for programs of temporal adjustment in the labor force if 10% (33% during the 2008 financial crisis) of their employees reduced their working hours by more than 10%. On average, the government subsidized 80% of the salary cost of furloughed employees, a proportion similar to that in Italy and the UK. The maximum duration of the subsidy was 21 months, but the normal time was 12 months. In Denmark, eligible firms were those that had fired 30% of employees or more, and the employees had to use up five vacation days before becoming eligible. The government subsidized 75% of the salary of employees who were laid off because of COVID-19. In France and Italy, eligible firms were those that want to totally or partially reduce the working time of their employees, and the subsidy extended for a maximum of 12 months in the case of France, and to the end of October 2020 in the case of Italy. In short, temporary workforce reduction programs, including ERTE in Spain, were being widely used throughout Europe. Although there are differences in the institutional design of the programs implemented, most share certain common characteristics: the corresponding procedures and eligibility requirements were similar, and the state covered a fairly high fraction of the salary that workers affected by a total or partial reduction in their work no longer received. Moreover, these plans and programs were relatively long-lasting (in the Spanish case, they have been extended until May 2021).

The measures adopted by the Spanish government intended to provide liquidity to firms, including delays in the payment of taxes (VAT, corporate), social security contributions, and rents, as well as loans and loan guarantees. Some loan and credit policy initiatives, to help firms and households overcome liquidity problems, were promoted by the ECB, while others were introduced under the initiative of the Spanish government. (A detailed list of government policies to provide liquidity appears in S1 Table of S1 Appendix). As noted, there is no information on whether firms in the sample requested and received subsidies, fiscal and loan guarantees, or government support, so we cannot analyze the effectiveness of public aid programs other than ERTE-aid.

### The effects of public aid

There are antecedents of empirical studies [9–11] analyzing the impact of temporary workforce reduction programs implemented during the Great Recession, and more recent papers investigating the effects of the COVID-19 pandemic on health and economic outcomes (see [12] for a review). This paper is in line with those that examine the direct (health of the working population) and indirect (social distance) effects of the pandemic in employment and in the working of labor markets [13–22]. Some of these papers use survey data, for example [14–16], as we do here, while others use register-statistical data, for example [17, 20, 22]. All conclude that the pandemic has had an important impact on the functioning of firms, including labor-based decisions that underline the notable differences among them.

The paper with data from Danish firms [15] is a case in point, but with some differences. The Danish data comes from a survey of firms with three or more employees, and examines the effect of ERTE-like liquidity support of government policies on firm employment decisions. The survey was launched in April and continued until June 1 (firms responded voluntarily to the survey and the response rate was 24%). Firms responded to the question of how many employees had been furloughed and/or laid off as a result of the pandemic, and firms that received public aid were also asked to indicate the number of employees that would have been laid off in the absence of the public aid. This counterfactual information provided by firms is not available in our study. The Danish study finds supporting evidence that firms with public aid laid off fewer employees than they otherwise would have, beyond those furloughed. The survey provides data on the actual number of employees laid off by firms in the first months of the pandemic, while the IAF survey, for Spain, asked firms about their prospective layoffs as the result of the pandemic in the coming six months, which would include the tourist season (important for employment in Spain).

The US survey [14] includes responses from 5,800 small firms in the US, to assess how COVID-19 was affecting their activities, as well as the likely impact of the stimulus bill, Coronavirus Aid, Relief, and Economic Security (CARES) Act in the continuation of the business. At the time of the survey, at the end of March and early April, 43% of the sample firms are temporary closed and, relatively to January, businesses, on average, had reduced employment by 40%. The results of the study also indicate that many firms were financially fragile and that cash holdings were positively associated with expectations of longer survival. At the time it was conducted, the study identified strong demand for Federal government subsidized aid and/or business loans. The expectation of survival in the near future increased when firms were informed about the establishment of the aid program.

The UK study [16], with data from the Understanding Society COVID-19 Survey, shows, among other things, that the government furlough scheme, by supporting household incomes and reducing income volatility, played an important role in maintaining aggregate consumption. However, the results were not quite as positive as they could have been. While 60% of individuals in the UK had had essentially no reduction of their household income (either no loss or less than 5%), 23% reported that household income had fallen by more than 20%., and that blacks and minority ethnic individuals, single-parent families, and those in the lowest long-term income quintile were the most affected by the reduction in personal income.

One research strategy to assess the potential economic damage of the pandemic has been through micro-simulations [19], covering extensive data at the company level (1 million companies, in 14 European countries), to evaluate the potential effectiveness of the two forms of job retention scheme in companies with liquidity constraints, the short-term work subsidies (STW) and the wage subsidy (WS). The survey found that STW subsidies were more effective in addressing liquidity problems in firms than WS schemes, because the former are targeted towards firms with greater financial difficulties. Wage Subsidy schemes potentially allow for larger reductions in labor costs for firms, while providing weaker protections for workers on reduced working hours.

The studies with US and UK data, as well as the micro-simulation study are different from the research reported here. The Danish study is the closest one, in terms of pros and cons. Spanish firms had the possibility to respond to the question of lay-offs taking place during the summer, when tourism and related activities are at their height, while firms did not respond regarding actual layoffs in the first month and a half of the pandemic, but rather about future intentions and prospects.

## Data and methods

### Characteristics of firms; sector and size

In April 2020, the Government of Aragon prepared a survey to assess the impact of the COVID-19 pandemic on the activities of firms in the region and, eventually, design and implement policies at the regional level to protect from severe damage. The survey was e-mailed to 5,000 firms, 10% of them being solo self-employed persons, registered in the database of the IAF as a result of participation and collaboration in previous business support programs (training, consulting, export, mentoring, financing). Firms chose to respond voluntarily and anonymously to the questionnaire through Google Forms, during the time period between 04/28/2020 and 05/08/2020. The final number of respondents was 796, a response rate of 16%, but in 69 cases the submitted forms were incomplete. Since decisions on furloughs and layoff of employees affected only the firms with employees, the 197 responses of solo self-employed were eliminated from the sample. Finally, 530 were included in the survey, 12% of the surveyed firms with employees. There are technical controversies around the relationship between response rates of surveys and the quality of the collected data [23–25]. Our S2 Appendix, provides additional information about key issues concerning the validity of the results, the representativeness of the responding firms, and the robustness of the findings. (More information on the questionnaire and on the firms can be found in the IAF web page (https://www.iaf.es/lim-covid19/index.php?subtipo=217#menu) and in [26]).

Descriptive information on the distribution of firms in the final sample, across economic sectors and size classes, together with the proportion that took ERTE aid in each case, is presented, respectively, in Tables 1 and 2. Table 1 provides complementary information on the distribution across economic sectors (one digit) of the population of firms in Aragon, and Table 2 provides information on the distribution of the population of firms and of all employees in Aragon and Spain, across size classes.

**Table 1:** Distribution of the sample of responding firms by economic sector.

		Survey IAF-COVID-19		Aragon Economy ^a^	Proportion of firms ERTE-aid
		N	% Firms	% Firms	
**A**	**Agriculture, Forestry and Fishing**	16	3.0	6.2	**6.3**
**C**	**Manufacturing**	140	26.4	16.3	**46.4**
**D-E**	**Energy and Water** ^**b**^	10	1.9	1.1	**40.0**
**F**	**Construction**	28	5.3	6.1	**39.3**
**G**	**Wholesale and Retail Trade; Repair of Motor Vehicles and Motorcycles**	62	11.7	14.7	**64.5**
**H**	**Transportation and Storage**	11	2.1	5.4	**36.4**
**I**	**Accommodation and Food Service Activities**	29	5.5	6.7	**79.3**
**J**	**Information and Communication**	23	4.3	1.9	**30.4**
**K**	**Financial and Insurance Activities**	9	1.7	1.6	**0.0**
**L**	**Real Estate Activities**	5	0.9	0.5	**60.0**
**M**	**Professional, Scientific and Technical Activities**	40	7.5	3.8	**20.0**
**O**	**Public Administration and Defense; Compulsory Social Security**	7	1.3	6.8	**0.0**
**P**	**Education**	34	6.4	4.1	**61.8**
**Q**	**Human Health and Social Work Activities**	22	4.2	9.3	**45.5**
**S**	**Other Service Activities**	94	17.7	15.6	**43.6**
	**Total**	**530**			**44.9**

^a^ Source: Own elaboration based on [27]

^b^ Energy and Water includes: Electricity, Gas, Steam and Air Conditioning Supply/Water Supply; Sewerage, Waste Management and Remediation Activities.

**Table 2:** Distribution of the sample of responding firms by firm size.

	Survey IAF- COVID-19	Aragon Economy 2019^a^	Spanish Economy 2019^b^		Proportion of firms ERTE-aid
	% Firms	% Firms	% Firms	% Employees	
**Micro (less than 10 employees)**	49.1	89.6	85.6	23.3	**41.5**
**Small (10 to 49 employees)**	31.3	8.7	12.1	21.1	**47.6**
**Medium (50 to 249 employees)**	13.2	1.3	2.0	16.7	**52.9**
**Large (250 or more employees)**	6.4	0.4	0.4	38.9	**41.2**
**Total**					**44.9**

^a^ Total number of firms in Aragon with employees 41,731

^b^ Source: DIRCE (Central Directory of Enterprises, INE)

There is a high correlation between the distribution of firms across economic sectors in the sample and the distribution of the population of firms in Aragon (Table 1), with some exceptions: the over-representation of Manufacturing firms and the under-representation of firms from the Primary and from the Transportation and Storage sectors. From column four of Table 1, 45% of the firms in the sample applied for ERTE aid, with significant variations across sectors. Human Health and Social Work Activities, Manufacturing, Other Service Activities, Energy and Water, and Construction, all contain a proportion of firms that applied for ERTE aid similar to the sample average. In the sectors Accommodation and Food Service Activities, Wholesale and Retail Trade, Repair of Motor Vehicles and Motorcycles, and Education, the proportion of firms that applied for ERTE aid is higher than the sample average.

The heterogeneity across economic sectors in the proportion of firms taking ERTE-aid reflects the different direct and indirect effects of the pandemic in certain production activities. The service sectors have been, in general, more affected than manufacturing and agriculture. Among the service sectors and related activities, those where teleworking is possible have been much less affected by the pandemic than those such as leisure, tourism, and retail—with the exception of food and other basic goods—and culture, where close interpersonal relations are inevitable. There is also a close correspondence between the proportion of firms that asked for aid across economic sectors in the sample, and the actual proportions of firms with ERTE-aid in the population of firms [28, 29].

Almost half of the firms in the sample, 49.1%, are micro firms (from 1 to 9 employees); 31.3% are small firms (from 10 to 49 employees); 13.2% are medium size (from 50 to 249 employees); and 6.4% are large (250 or more employees) (Table 2, column one). The 530 firms with employees in the sample represent 1.3% of the total number firms with employees in Aragon (41,731). Compared with the distribution of the population of firms in Aragon and Spain across size classes, in the sample data the micro firms are under-represented and the rest of the size classes are over-represented. Notice, however, that the distribution of employees across size classes is more homogeneous than the distribution of firms (only data for Spain is available for this variable). The final column of Table 2 shows the proportion of firms taking ERTE-aid in each size class. Proportions range between 41.2% among large firms, and 52.9% among the medium size ones. Remarkably, the proportion of firms that asked for ERTE-aid is not that different across size classes.

### Prospects on activities, revenues, and employment

Table 3 summarizes the responses of firms to questions about their prospects of discontinuing activity, fall in revenues from sales, and loss of employment within the next six months (till the end of September), by size classes and in the subsamples of firms that asked for ERTE aid and those that did not. Almost half of the respondents, 46%, did not discontinue their activity due to the COVID-19. Around two thirds, 67.3% either did not stop their activities, or expected to reopen within less than two months; 11% expected to recover their business after six months. Conditioned to reopening in the expected time, 13.6% anticipated a reduction in sales lower than 5%, while more than one fourth, 25.8%, anticipated a reduction above 40%. The median firm in the sample anticipated a reduction in sales as a result of the pandemic, in the next six months, between 25% and 40%.

**Table 3:** Distribution of the sample of responding firms by their prospects for the next six months of activities, revenues, and employment.

	Survey IAF-COVID-19						ERTE-aid (%)		
	Total		Size classes (%)						
	N	%	Micro	Small	Medium	Large	Distribution of firms, no aid	Distribution of firms aid	Proportion of firms aid
**Activity interruption prospects**									
Not discontinued	244	46.0	39.2	50.6	54.3	58.8	69.5	17.2	**16.8**
Less than 2 months	113	21.3	23.1	20.5	14.3	26.5	13.4	31.1	**65.5**
Between 2 and 6 months	116	21.9	22.7	22.	25.7	5.9	12.7	33.2	**68.1**
More than 6 months	57	10.8	15.0	6.6	5.7	8.8	4.5	18.5	**77.2**
**Total**	**530**								**44.9**
**Revenue reduction prospects**									
More than 40%	137	25.8	35.8	17.5	17.1	8.8	15.4	38.7	**67.2**
Between 25% and 40%	157	29.6	30.0	28.9	34.3	20.6	22.9	37.8	**57.3**
Between 10% and 25%	106	20.0	15.4	25.3	22.9	23.5	22.6	16.8	**37.7**
Between 5% and 10%	58	10.9	6.9	16.3	14.3	8.8	15.1	5.9	**24.1**
Up to 5%	72	13.6	11.9	12.0	11.4	38.3	24.0	0.8	**2.8**
**Total**	**530**								**44.9**
**Employment reduction prospects**									
More than 20%	113	21.3	28.9	11.4	18.6	17.7	12.0	32.8	**69.0**
Between 10% and 20%	71	13.4	13.1	15.1	12.8	8.8	10.6	16.8	**56.3**
Between 5% and 10%	64	12.1	6.9	18.7	20.0	2.9	9.2	15.5	**57.8**
Up to 5%	64	12.1	3.8	17.5	22.9	26.5	13.7	10.1	**37.5**
0% (Will be kept)	218	41.1	47.3	37.3	25.7	44.1	54.5	24.8	**27.1**
**Total**	**530**								**44.9**

The prospects for employment reduction, bottom part of Table 3, indicate that 41.1% of the firms do not expect changes in their employment, whereas 60% expect to reduce their employees within the next six months. Half of the firms in the sample estimate and employment reduction of 5% or more, and the other half estimate that their reduction in employment will be less than 5% (for the median firm, the expected loss in employment is zero). Around 21% of the firms in the sample expect a reduction in employment above 20%. Overall, smaller firms exhibit the worst prospects.

Sample average reductions, calculated from the center of the classes in the respective range of values considered in the survey, are 27.2% reduction in revenues from sales, and 8,5% reduction of employment (an elasticity of employment reduction to an output fall of 31.1%). The survey does not provide information on the number of furloughed employees or on the number of employees laid off if the ERTE-aid had not been available.

### Research questions and method

This section addresses three main questions: i) Did the firms that asked for ERTE-aid were those targeted by the public aid program? ii) Did the ERTE aid program contributed to reduce the sensitivity of employment to the prospects on activities and fall in revenues? iii) Did the ERTE aid program contribute to reduce the expectation of the number of employees laid off, beyond those employees that were furloughed?

The answer to the first question will come from the results of estimating a probit model with dependent variable 1 if the firm received ERTE aid and 0 otherwise, and explanatory variable the characteristics of firms in the sample. The answer to the question would be yes if the probability of taking ERTE aid is higher among firms with worst prospects on activity and fall in revenues than among firms with relatively better prospects, controlling for size and sector. The ultimate goal of the ERTE program is to reduce the sensitivity of employment to external aggregate negative demand and activity shocks. In the context of question two, we estimate an ordered probit model with dependent variable the ordered expectations of employees to be laid off and explanatory variables the characteristics of firms. The estimation is done separately among firms that ask for ERTE aid and among firms that did not ask for aid. For the goals of the program being fulfilled, the estimated sensitivity of prospects of employees’ lay off to the prospects on reduction of activity and fall in sales is lower in the sample of ERTE aid firms than in the sample of no ERTE aid.

Finally, the answer to question three requires estimating the values of the counterfactual variable “predicted values of prospects of employees laid off for each firm in the sample, if the firm had responded with layoffs to prospects on activity and fall in revenues, in the same way that did, on average, the firms that did not ask for ERTE aid”. Next with a test of difference in difference, we compare the difference between estimated and observed prospects of laid offs in the sample of firms that took ERTE aid and in the sample of firms that did not take aid. If the difference was lower in the sample of firms with ERTE aid then the aid program would have contributed to save jobs beyond those of the furloughed employees.

## Results of the empirical analysis

### The decision of firms to take government aid (ERTE) or not

Evidence from Table 3 suggests that most of the firms that do not ask for ERTE aid, close to 70%, do not expect to discontinue their activities as a result of the pandemic, while, among those firms that applied, the median of time that they expect to be affected is between two and six months. With respect to revenue, only 2.8% of the firms with expectation of a reduction in revenues of 5% or less took ERTE-aid, while 57% to 67% of firms that anticipated a decrease in revenues of 25% or more took the aid. The distribution of firms across levels of revenues reduction is fairly even within the firms that did not take ERTE aid (with a median in the range of prospects of revenue declines of between 10% and 25%), while in the group of firms that did take ERTE aid more than three fourths of the firms anticipated a fall in revenues equal to or higher than 25%.

**Fig 1:**
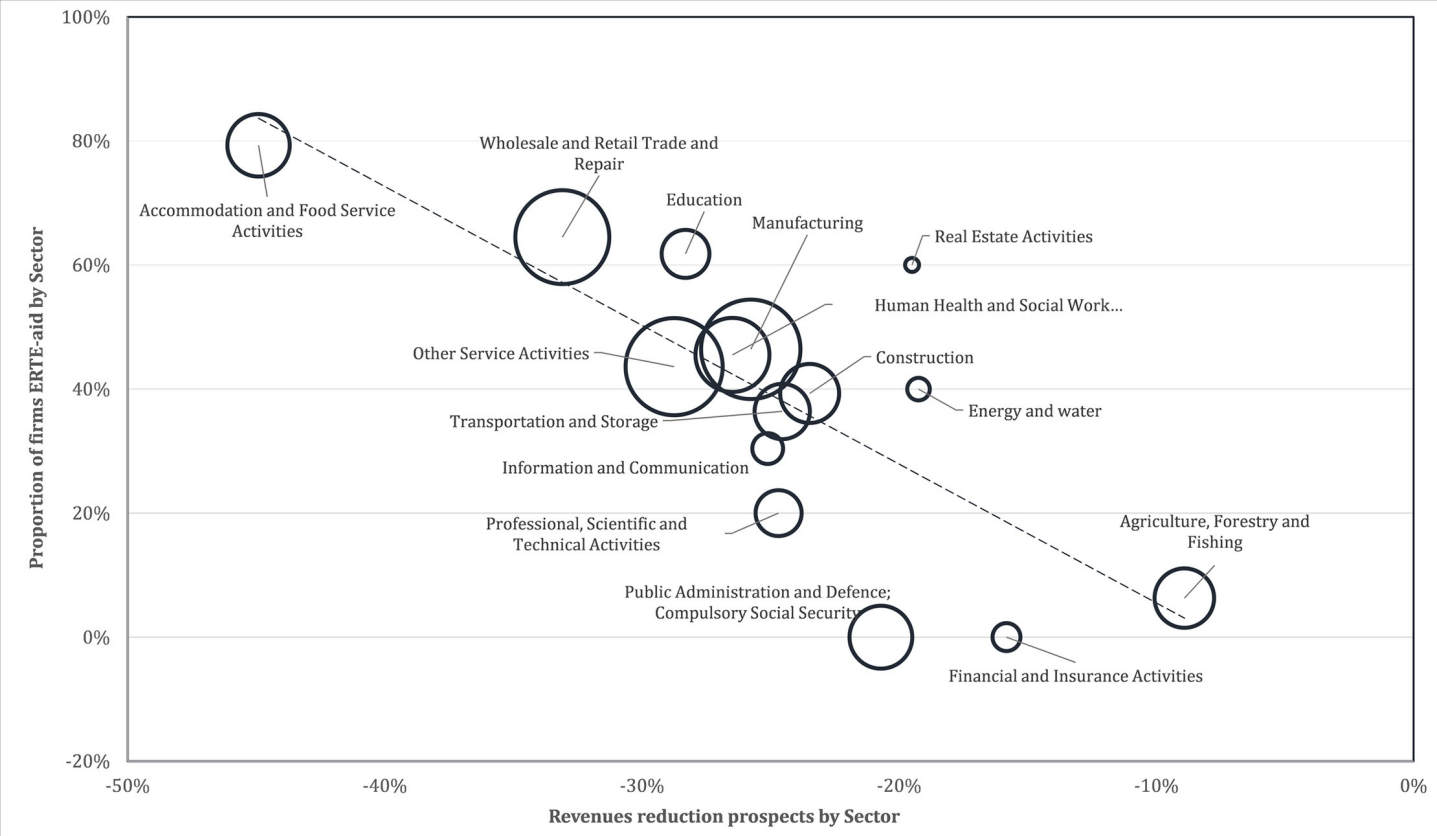
Proportion of firms with ERTE-aid and revenues reduction prospects.

Fig 1 plots the relationship between the expected fall in revenue for the next six months and the proportion of firms that took ERTE-aid, across economic sectors. Each circle represents a sector, with the size of the circle being proportional to the relative number of firms in the sector, over the total number of firms in the sample. The prospects of falls in revenue show significant variation across sectors, consistent with the heterogeneous effects of the pandemic. There is a clear correlation between the expected reduction in revenues across sectors and the proportion of firms that took ERTE aid, with greater expected falls in revenue associated with a greater proportion of firms applying for ERTE aid. Firms in Accommodation and Food Services Activities, with an expected fall in their sales revenues of 45% on average, are the most likely to demand aid, with 80% of those firms responding in this way. On average, a prospective 25% decrease in revenue is associated with a 50% likelihood of applying for aid.

Therefore, the descriptive information indicates that the prospects on activity and fall in revenues are worst among firms that asked for ERTE aid than among firms that did not ask for aid. The multivariate statistical analysis will consist on the estimation of a probit model of the decision to take ERTE aid, or not. The dependent variable takes the value of 1 if the firm takes the aid and 0 otherwise and the explanatory variables include, the economic sector, firm size, time till reopening activities, and fall in revenues from sales. The results of the estimation appear in Table 4. The null hypothesis that the coefficients of all explanatory variables are equal to zero is rejected with p<1% (χ^2^ = 247.57). For separate groups of explanatory variables, the joint χ^2^ test of the null hypothesis that all coefficients are equal to zero is rejected at p<1% for variables of size, and prospects of reduction of activity and of fall in sales revenues. Among the sector variables, the most statistically significant estimated coefficient is that of the Wholesale and Retail Trade, and Repair of Motor and Vehicles sector. From the estimated coefficient, controlling for the rest of explanatory variables, the probability of ERTE-aid is 25.9% higher in the sector of Wholesale and Retail Trade, and Repair of Motor and Vehicles than in the sector of Professional, Scientific and Technical Activities (omitted variable).

**Table 4:** Probit model on the decision to take ERTE-aid.

	Coef. (Std. Err.)	Marginal effect
**Sector**		
Agriculture, Forestry and Fishing	-0.124 (0.676)	-0.031
Manufacturing	0.552 (0.318) *	0.145*
Energy and water	0.579 (0.538)	0.153
Construction	0.340 (0.397)	0.089
Wholesale and Retail Trade; Repair of Motor and Vehicles	0.988 (0.343) ***	0.259***
Transportation and Storage	0.446 (0.629)	0.117
Accommodation and Food Service Activities	0.780 (0.406) *	0.205*
Information and Communication	0.208 (0.440)	0.054
Financial and Insurance Activities	-	-
Real Estate Activities	0.845 (0.677)	0.222
Public Administration and Defense; Compulsory Social Secur.	-	-
Education	0.780 (0.388) **	0.206**
Human Health and Social Work Activities	0.388 (0.412)	0.102
Other Service Activities	0.421 (0.327)	0.110
χ^2^(12)	14.88	
**Size**		
Small	0.645 (0.169) ***	0.159***
Medium	0.846 (0.225) ***	0.205***
Large	1.273 (0.375) ***	0.298***
χ^2^(3)	25.47***	
**Activity interruption prospects**		
Less than 2 months	1.383 (0.184) ***	0.411***
Between 2 and 6 months	1.210 (0.186) ***	0.360***
More than 6 months	1.509 (0.245) ***	0.446***
χ^2^(3)	78.12***	
**Revenues reduction prospects**		
Between 25% and 40%	-0.242 (0.175)	-0.071
Between 10% and 25%	-0.567 (0.209) ***	-0.169***
Between 5% and 10%	-1.087 (0.265) ***	-0.316***
Up to 5%	-2.145 (0.414) ***	-0.520***
χ^2^(4)	37.04***	
Constant	-1.317 (0.323) ***	
Observations	514	
χ^2^(22)	247.57***	

Note

*Statistically significant at the .10 level

** at the .05 level

*** at the .01 level. The reference category for sector: Professional, Scientific and Technical Activities; for size: Micro; for activity prospects: Not discontinued; for revenues reduction prospects: More than 40%.

The probability of taking ERTE-aid increases with the size of the firm. The probability of ERTE aid is 15.9% higher among small firms, and 29.8% higher among large firms, compared with the probability of taking aid among micro firms (omitted variable). The probability of taking ERTE aid is about 40% higher among firms that expect having to interrupt their activity than among those that do not expect interruption, and the difference in probability increases moderately with the duration of the interruption. Finally, the likelihood of taking ERTE aid among firms with modest expected falls in revenues (up to 5%) is more than 50% lower than the likelihood of taking ERTE aid among firms that expect a fall in revenues of 25% or more.

Overall, the multivariate analysis confirms that firms not in distress from COVID-19 are less likely to take government ERTE-aid than firms in distress. And, within the distressed firms, the probability of asking for ERTE increases with the magnitude of the distressed, both in terms of prospects of duration of the interruption of activity and prospects of fall in revenues from sales. Therefore, the ERTE aid program achieved the first goal of being attractive for firms with worst prospects from the pandemic. The statistically significant sector effects limited to one sector, controlling for the rest of variables, suggests that in the eligibility of firms for government aid the characteristics of the firm should weight more than the sector where it belongs.

### The effect of ERTE-aid on labor adjustments and on job loss expectations

#### The adjustment of employment to the fall in activity as the result of the pandemic

Among ERTE aid firms the adjustment of employment in response to external shocks, pandemic related shocks in this case, can be done with decisions on the number of employees furloughed and on the number of employees laid off. Among the no ERTE aid firms the only labor adjustment variable is the layoff of employees. Therefore, the sensitivity of layoffs of employees to the prospects on activity and revenues should be higher among non ERTE aid firms than among ERTE aid ones. Table 5 shows the results of the multivariate analysis, ordered probit models, of prospects of job losses as a function of characteristics of firms and sector variables, separated for firms that take ERTE-aid for those that don’t. The dependent variable is ordered from higher to lower proportion of employees expected to be laid off: more than 20%, between 10 and 20% laid off, between 5% and 10% laid off, less than 5%, and no laid offs. Thus, a positive (negative) estimated coefficient will indicate that the variable contributes to reduce (increase) the prospect of loss of employment. The explanatory variables are sector, size, and prospects for activity and for reduction in revenues.

**Table 5:** Ordered probit estimation of anticipated employment reduction for firms with ERTE-aid and for firms without ERTE aid.

	Not ERTE-aid	ERTE-aid	χ^2^ for
	Coef. (Std. Err.)	Coef. (Std. Err.)	difference
**Sector**			
Agriculture, Forestry and Fishing	-0.590 (0.435)	-0.521 (1.126)	0.01
Manufacturing	-0.207 (0.287)	-1.052 (0.466) **	2.04
Energy and water	-0.105 (0.557)	-0.677 (0.701)	0.57
Construction	-0.642 (0.366) *	-1.288 (0.561) **	0.97
Wholesale and Retail Trade; Repair of Motor and Vehicles	-0.350 (0.344)	-0.583 (0.476)	2.15
Transportation and Storage	-0.510 (0.548)	-1.097 (0.709)	0.40
Accommodation and Food Service Activities	-1.296 (0.599) **	-1.634 (0.508) ***	0.19
Information and Communication	-0.416 (0.380)	-0.768 (0.604)	0.23
Financial and Insurance Activities	-0.034 (0.554)	-	-
Real Estate Activities	-0.386 (0.853)	-0.399 (0.858)	0.00
Public Administration and Defense; Compulsory Social Sec	0.460 (0.591)	-	-
Education	-0.396 (0.425)	-1.136 (0.502) **	1.10
Human Health and Social Work Activities	-0.431 (0.424)	-1.511 (0.572) ***	2.13
Other Service Activities	-0.326 (0.290)	-0.969 (0.472) **	1.14
χ^2^(14/12)	10.77	19.93*	8.70
**Size**			
Small	-0.210 (0.181)	0.006 (0.184)	0.76
Medium	-0.344 (0.257)	-0.224 (0.231)	0.12
Large	-0.676 (0.330) **	-0.202 (0.354)	1.10
χ^2^(3)	5.06	1.37	1.46
**Activity interruption prospects**			
Less than 2 months	-0.209 (0.223)	-0.015 (0.235)	0.34
Between 2 and 6 months	-0.411 (0.224) *	0.026 (0.238)	1.52
More than 6 months	-1.397 (0.370) ***	-0.123 (0.265)	6.29**
χ^2^(3)	15.54***	0.44	6.51*
**Revenues reduction prospects**			
Between 25% and 40%	0.400 (0.232) *	0.647 (0.184) ***	0.54
Between 10% and 25%	1.153 (0.248) ***	1.076 (0.242) ***	0.05
Between 5% and 10%	1.241 (0.275) ***	1.617 (0.352) ***	1.00
Up to 5%	2.219 (0.309) ***	1.325 (0.775) *	4.00**
χ^2^(4)	63.33***	31.83***	11.38**
Observations	292	238	
χ^2^(24/22)	135.37***	68.15***	43.48***

Note

*Statistically significant at the .10 level

** at the .05 level

*** at the .01 level. The reference category for sector: Professional, Scientific and Technical Activities; for size: Micro; for activity prospects: Not discontinued; for revenues reduction prospects: More than 40%.

The estimations from the two samples are jointly statistically significant, although the null hypothesis of no joint statistical significance is rejected with a greater likelihood ratio in the subsample of firms with no ERTE aid than in the subsample of ERTE aid firms, χ^2^ = 135.37 and χ^2^ = 68.15, respectively. Moreover, the null hypothesis of equal regression coefficients of the two probit models is rejected, χ^2^ = 43.48 (p<1%). Therefore, overall, the association between prospects of employment and prospects on activity and revenues from sales is higher in the subsample of no ERTE aid firms than among ERTE aid firms, as expected. Looking at differences of estimated coefficients across individual explanatory variables, they concentrate, in a statistically significant way, in two variables, prospects of reopening after having stopped activities for 6 months or more, and prospects for a fall in revenues of 5% or less in the next six months.

It is remarkable that among firms with no ERTE aid, the expectations of the adjustment of employment respond only, in a statistically significant way, to variables of prospects of stopping/initiating activities (with a greater reduction of employment in firms that expect to reinitiate their activities after six months or more), and prospects of the fall in revenues (with higher expected layoffs as the fall in revenues worsens), and not to characteristics of the sector of activity or the size of the firm.

**Fig 2:**
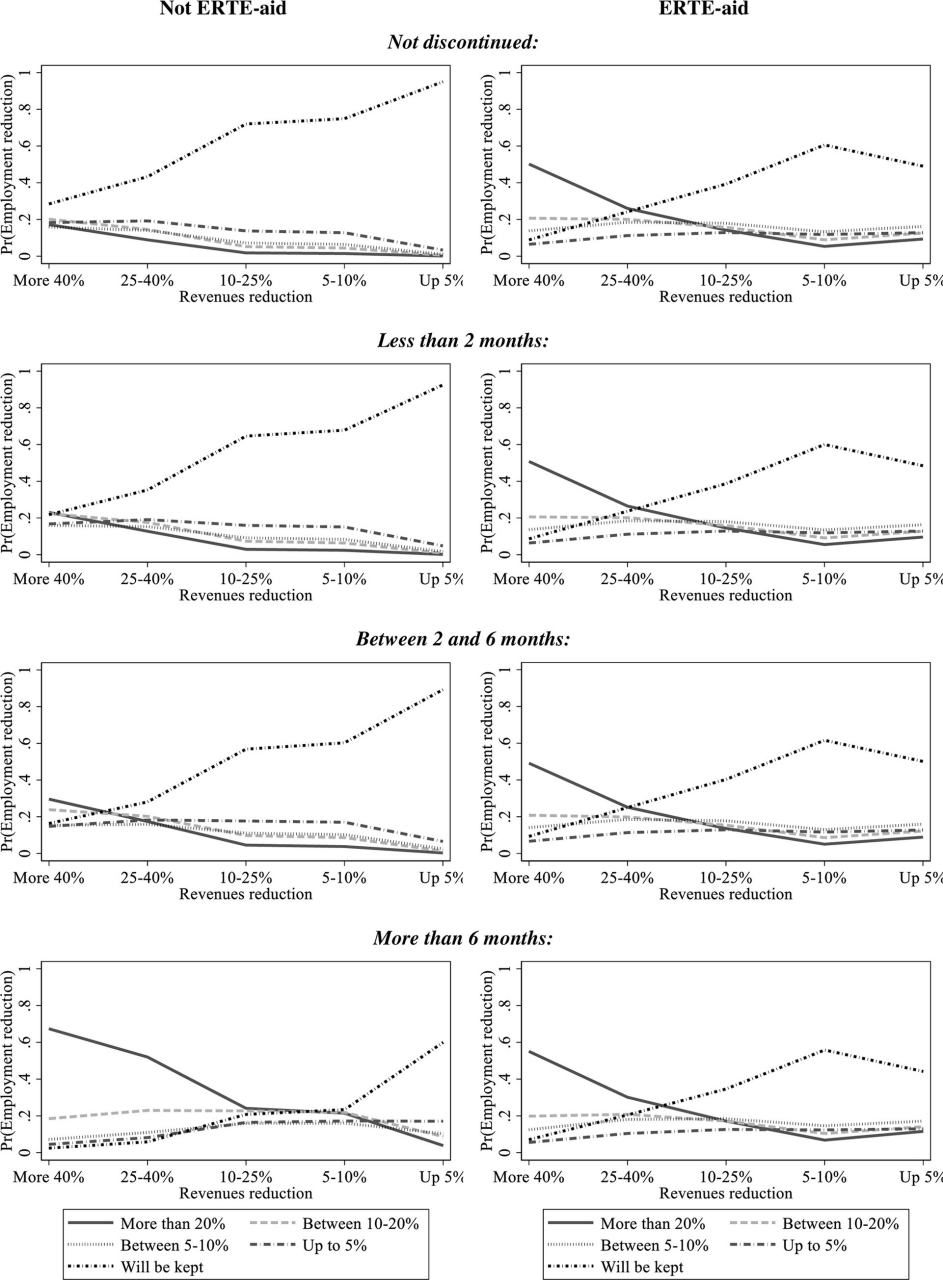
Predicted probability of layoffs from revenue reduction and activity prospects for firms with or without ERTE-aid.

The results of Table 5 are now complemented with a graphical comparison of the sensitivity of expectations of job losses to the prospects of activity and fall in revenues among firms that take aid, compared with the sensitivity of those that do not take it. Fig 2 shows the predicted values of the probability of layoffs as a function of prospects of the fall in revenues, separated for firms that take aid and for firms that do not, and separated for firms with different expectations of reinitiating activities. The predicted values are calculated with the coefficients estimated in Table 5 for the respective group of firms.

Among the firms that do not take ERTE-aid, better prospects for the evolution of revenues (from reductions of more than 40% to reductions of 5% or less) increase the probability of no layoffs (the employment will be maintained), and decrease the probability of layoffs affecting 20% of employees. This happens for all prospects of the evolution of activity, i.e. among firms that expect not to discontinue their activities, and those that expect to discontinue their activities and reopen after six months or more. Notice also that as the prospect of reopening dims, the probability of no layoffs moves downwards, whereas the probability of layoffs affecting more than 20% of employees moves upwards. The displacements, up and down, in the estimated probability values, with respect to the case where firms expect that activities will not be discontinued, is particularly high among those firms that do not take ERTE-aid and anticipate being inactive for six months or more. For instance, the probability that a firm expecting a reduction in revenues of at least 40%, would reduce employment by 20% or more, is around 0.2 among firms that expect not to interrupt their activities, and rises to 0.66 in firms expecting a resumption of activities within six months.

Among firms that take the ERTE-aid, the probabilities of layoff are not sensitive to the prospects of falling revenues from sales, and this result holds across all prospects of stopping/reinitiating activities. When comparing the estimated probability that layoffs will affect at least 20% of employees in firms with or without aid, that probability is higher in the sample of firms taking aid than in that of firms not taking aid. At the same time, the estimated probability of no layoffs is lower in the sample of firms with aid than in the sample of firms with no aid. This is true for all cases, except in the subsample of firms that expect to resume activities after six months or more, where the proportion of firms that expect high layoffs is higher in non-aid firms than in aid firms.

All these results explain, first, that, overall, the 10.75% expected proportion of employees laid off in the subsample of firms with ERTE aid is higher than the 6.6% expected proportion in the subsample of firms with no ERTE aid. Second, that among the firms with no ERTE aid, there is a subset of firms with prospects of reinitiating after a long period, more than six months, for which taking the ERTE aid is simply not worthwhile.

#### Differences in expected layoffs of employees in ERTE and no-ERTE aid firms

The greater expected proportion of employees laid off in the subsample of firms with ERTE aid than in the subsample of no ERTE aid, may question the effectiveness of the aid program, since the possibility of furloughing employees does not impede that ERTE aid firms expect to lay off higher proportion of employees than the no ERTE aid ones. However this conclusion may not be right because the direct comparison of expected proportions of employees laid off in the two samples, ignores the different prospects of activity and revenues among firms that condition the decision to apply for aid or not. Moreover, it is unknown what would had happened in terms of job losses without the ERTE aid program. This section provides an assessment of the potential jobs saved by the ERTE aid program.

The first analysis examines the results of comparing expected layoffs of employees between ERTE aid and no ERTE aid firms, when not controlling and when controlling for differences in the characteristics of firms. For this purpose, Table 6 shows the results of estimating a univariate and a multivariate ordered probit models. The dependent variable is the ordered range of proportions of employees laid off, from high to low proportions, as in Table 5. The dummy variable *ERTE-aid* that takes the value of 1 if the firm applied for ERTE aid and zero otherwise is included as explanatory variable in all estimations. More formally, the empirical ordered probit model to be estimated is formulated as follows:

$$Prob(Y_{i}=j)=F(\delta \;ERTE‐aid_{i}+\beta \;X_{i})$$ (1)

**Table 6:** Ordered probit estimation: ERTE policy evaluation.

	(1) Observed data	(2) Observed data	(3) Observed data	(4) Counterfactual / Observed data	χ^2^ for difference (3)-(4)
	Coef. (Std. Err.)	Coef. (Std. Err.)	Coef. (Std. Err.)	Coef. (Std. Err.)	
**ERTE-aid**	-0.778 (0.098) ***	-0.664 (0.104) ***	-0.133 (0.125)	-0.159 (0.108)	0.04
T * ERTE-aid				0.123 (0.106)	
**Sector**					
Agriculture, Forestry and Fishing		-0.055 (0.362)	-0.655 (0.394) *	-0.916 (0.290) ***	1.84
Manufacturing		-0.456 (0.222) **	-0.477 (0.234) **	-0.317 (0.175) *	1.11
Energy and water		-0.104 (0.400)	-0.261 0.413	-0.191 (0.325)	0.12
Construction		-0.662 (0.284) **	-0.787 (0.296) ***	-0.905 (0.222) ***	0.37
Wholesale and Retail Trade; Repair of Motor and Vehicle		-0.375 (0.244)	-0.272 (0.256)	-0.441 (0.189) **	0.95
Transportation and Storage		-0.483 (0.391)	-0.558 (0.418)	-0.581 (0.317) *	0.01
Accommodation and Food Service Activities		-1.585 (0.307) ***	-1.240 (0.322) ***	-1.553 (0.253) ***	3.74*
Information and Communication		-0.350 (0.305)	-0.422 (0.317)	-0.338 (0.238)	0.14
Financial and Insurance Activities		0.023 (0.473)	-0.186 (0.525)	-0.033 (0.408)	0.39
Real Estate Activities		0.055 (0.554)	-0.133 (0.581)	0.060 (0.450)	0.36
Public Administration and Defense; Compulsory Social S		-0.063 (0.512)	0.119 (0.549)	0.264 (0.432)	0.50
Education		-0.573 (0.273) **	-0.613 (0.288) **	-0.554 (0.211) ***	0.10
Human Health and Social Work Activities		-0.810 (0.310) ***	-0.828 (0.322) ***	-0.711 (0.236) ***	0.36
Other Service Activities		-0.513 (0.226) **	-0.476 (0.239) **	-0.510 (0.180) ***	0.05
χ^2^(14)		37.63***	24.30**	64.87***	33.00***
**Size**					
Small		0.208 (0.117) *	-0.093 (0.125)	-0.213 (0.093) **	2.40
Medium		0.021 (0.156)	-0.299 (0.165) *	-0.261 (0.123) **	0.14
Large		0.195 (0.213)	-0.492 (0.233) **	-0.359 (0.177) **	1.18
χ^2^(3)		3.75	6.42*	8.61**	6.06
**Activity interruption prospects**					
Less than 2 months			-0.156 (0.148)	-0.351 (0.110) ***	4.64**
Between 2 and 6 months			-0.195 (0.152)	-0.453 (0.111) ***	6.83***
More than 6 months			-0.512 (0.193) ***	-0.828 (0.142) ***	7.17***
χ^2^(3)			7.04*	37.23***	11.08**
**Revenues reduction prospects**					
Between 25% and 40%			0.571 (0.140) ***	0.522 (0.101) ***	0.26
Between 10% and 25%			1.166 (0.167) ***	1.140 (0.122) ***	0.05
Between 5% and 10%			1.424 (0.204) ***	1.476 (0.153) ***	0.17
Up to 5%			2.217 (0.248) ***	2.179 (0.196) ***	0.09
χ^2^(4)			102.94***	181.48***	1.21
Observations/Firms	530/530	530/530	530/530	1060/530	
χ^2^ for global model	62.85***	106.50***	244.71***	542.53***	79.40***

Note

*Statistically significant at the .10 level

** at the .05 level

*** at the .01 level. The reference category for sector: Professional, Scientific and Technical Activities; for size: Micro; for activity prospects: Not discontinued; for revenues reduction prospects: More than 40%.

Firms are indexed by *i*; vector *X*_*i*_ includes the explanatory variables other than *ERTE-aid*. The results of the estimations for different vectors of explanatory variables appear in columns (1) to (3) of Table 6.

The negative and statistically significant estimated coefficient of the *ERTE-aid* variable in column (1) confirms that, with no controls, the expected proportion of employees laid off is greater in ERTE aid firms (recall that the dependent variables are ordered from high to low proportion of expected layoffs). The estimation in column (2) controls for the characteristics of firm sector and size, and the estimated coefficient of the *ERTE-aid* variable continues to be negative and statistically significant. In column (3) the estimation includes additional controls, i.e. prospects for stopping/reinitiating activities and prospects for falls in revenue. The coefficient of the *ERTE-aid* variable is now not statistically significantly different from zero: when considering the different prospects of firms about the impacts of the pandemic on their respective activities, the differences in prospects for lay-offs in the two subsamples disappear. In other words, the worst prospects for the impact of the pandemic on their activity that lead firms to apply for ERTE aid, are the same as those that determine the expectation of ERTE aid firms to have more job losses than the firms with no ERTE aid.

Although the changes in the estimated coefficient of the *ERTE-aid* variable with controls, from negative and significant to non-significant, inform that asking for ERTE aid is not sufficient to assess the effect of ERTE on layoffs, the ideal way to respond to the question about the effects of ERTE aid on layoffs would be by comparing the layoffs with ERTE with those in the case of no ERTE. This counterfactual analysis is not possible, nor do we have direct information from the firms on their assessment of what they would have done in the hypothetical situation where the ERTE aid had not been available [15].

A second-best exercise consists on artificially constructing a counterfactual in a way similar to what is a common practice among national governments and international organizations, particularly the World Bank, to assess the causal effects of economic policy interventions (see [30] or [31]). The method goes as follows: first, estimate the model that explains the expected losses in employment with data only of the firms that did not apply for ERTE-aid. Second, use the coefficients of the estimated model to predict the losses in employment for firms in the sample, taking as predictors the values of the explanatory variables of the respective firm. Third, statistically compare the predicted losses of employment with the actual ones for all firms in the sample. The key assumption for the validity of the methodology is that under similar circumstances, all firms would have had similar prospects of layoffs (see [32, 33] for somewhat similar approaches).

Pooling the observed and predicted values of the dependent variable, the empirical model to be estimated is the following:

$$Prob(Y_{iT}=j)=F(\delta_{1}\;ERTE‐aid_{i}+\delta_{2}\;T*ERTE‐aid_{i}+\beta \;X_{i})$$ (2)

Firms are indexed by *i* and *T* is a dummy variable that takes the value of *T* = 1 when the value of the variable is the observed one, and 0 when it is the predicted one—the counterfactual- and so there are two observations per firm, one with the observed value of the dependent variable, *Y*_*i1*_ (with *T* = 1), and the other with the predicted value, *Y*_*i0*_ (with *T* = 0). *ERTE-aid*_*i*_ is the same dummy variable as above; and *X*_*i*_ is a vector of the rest of explanatory variables, sector size, and prospects for activities and revenues. The coefficient *δ*_*1*_ will indicate the difference in the probability of the level of layoff between firms that take the aid and firms that do not; the coefficient *δ*_*2*_ is an estimate of the difference in difference, that is the average differential effect on the value of the dependent variable of the policy variable, ERTE-aid in this case, for those firms that take the aid. For the interpretation of the results, it should be kept in mind that the dependent variable is ordered from higher prospect for loss of employment (more than 20%) to lower prospect (no loss at all). Thus, a positive (negative) estimated coefficient will indicate a decrease (increase) in the projected proportion of employees affected by layoffs.

The results of the estimation of model (2) appear in the fourth column of Table 6. The coefficients of the *ERTE-aid* and T*ERTE-aid variables are not statistically significant different from zero. The non-significant estimated coefficient of the *ERTE-aid* variable indicates that there are no statistical differences in the average layoffs of firms with and without ERTE aid, including the prospects for layoffs by firms in the surveys and those predicted in the counterfactual exercise. The non-statistically significant coefficient of T*ERTE-aid indicates that the ERTE aid had no differential effect on the decision of layoffs by the firms that took the aid, compared with the decision that the firms with no ERTE aid would have made under similar circumstances.

## Conclusion

The COVID-19 virus has disrupted social life and economic activity around the world, with consequences for employment and labor policies of firms across all sizes. This paper examines the responses of a sample of 530 firms located in Aragon (Spain) to a survey (response rate of 12%) with questions about their prospects for how COVID-19 would affect their business activity, in the short and mid-term. The survey was conducted at the end of April and first week of May 2020, one month and a half after the Spanish government declared the state of alarm. The study has two main goals. First, to assess how informative about the impact of the pandemic in the months ahead were the responses of the firms to the survey, and month after the declaration of the state of alarm, and eventually how useful the information collected could be for policy decisions. Second, considering the informal and unorthodox ways that the timely information was collected, if, with what we know one year later, the information on prospects collected in April 2020 has proven to be sufficiently close to what actually happened in the Spain and Aragon’s economies during the rest of the year.

The first message sent by the firms to the Government in April 2020, was that the COVID-19 crisis would have a very negative impact. Close to one third of the firms in the sample expected to discontinue their activity for more than two months. The median firm anticipated a decline in revenue from sales was between 25% and 40% for the next six months. And, even though 45% of the firms in the sample took ERTE-aid (the government subsidy of labor costs of furloughed employees), the firms in the sample estimated an average expected loss of employment in the following six months of 8.5%.

The second result, related to the research questions, is whether firms responded to the ERTE aid as intended by the policy maker. Firms with the worst prospects, in terms of having to stop their activities and wait longer to resume, and in terms of a greater than anticipated fall in revenue from sales, were more likely to apply for ERTE aid than firms otherwise. With the offered aid, firms had the possibility of adjusting their active labor force in response to the fall in demand, either through furloughed employees, applying for aid, or through layoffs, if they did not ask for aid. This is what we observe in the data: the sensitivity of the prospects for layoffs to the prospects for business activity (stop/reinitiate, fall in revenues) was more intense in the subsample of firms that did not ask for ERTE aid than among the firms that did.

The data also show that the average expected loss of employment in the subsample of firms that applied for ERTE aid is larger than the average expected loss of employment in the subsample of no ERTE firms. This result may suggest that the aid policy was not being as effective as expected in preventing the destruction of jobs. However, when controlling for the different characteristics of firms, the averages of the anticipated loss of employees in the two subsamples are no longer statistically significantly different from zero. The main determinant of the change in the result was controlling for the prospects of activities. Controlling only for size and sector of the firm the difference continued being statistically significant. The policy message for the government was that aid policies should be based on the situation of individual firms, and not conditioned on the sector or on the size of the firm.

The ideal test of the effectiveness of the public aid policy would have required observing what would have happened if the aid had not been introduced (counterfactual analysis). This is not possible and instead, our paper presents a (imperfect) counterfactual exercise. The exercise consists on comparing, across all firms in the sample, the observed prospects for layoffs with those predicted assuming that, for all firms in the sample, the relationship between prospects of layoffs and prospects of activity is that estimated in the subsample of no ERTE aid firms. The difference in difference estimation with observed and predicted data does not reject the null hypothesis that, under similar circumstances, the no ERTE aid firms had the same prospects for layoffs as the ERTE aid firms. Thus, the ERTE aid saved the jobs of the furloughed employees, but no more, and no less.

A fair question, given the way the survey data were collected, is about representativeness of the sample of firms that responded the survey, and the quality of the responses. When firms answered the survey, the responses could only be in terms of expectations or intentions for the coming months, not what they would actually do. In fact, the ultimate effect of the pandemic could well differ from what was anticipated in April-May of 2020 (S2 Appendix provides more detail on this issue). Somewhat surprisingly, the sample of respondents covers reasonably well all economic sectors and size classes, although with under-representation of micro firms (other surveys, for example [15], restricted the population of firms to those with a minimum number of employees, i.e. 3, while the IAF sample was open to all firms with and without employees). Second, the evidence presented in the S2 Appendix indicates that, in terms of aggregates for sector and size of firms, the prospects of the firms of the sample, in April-May 2020, about the evolution of revenues and employment in the next six months turned out to be quite close to the observed aggregates one year later. This conclusion is important, because we conjecture that the way that the Government of Aragon collected firm-level data to assess the response of firms to the pandemic, will be more common in the near future (the Bank of Spain did something similar in November 2020). Having timely data is positive for the timing of policy decisions, but there must be some confidence in the representativeness and reliability of the collected data.

Considering that the ERTE-aid program was a novelty among public policies implemented in Spain, it may also be of interest to assess its potential efficacy, comparing what happened during the Great Recession to what happened this time. The number of workers employed during the second and third quarters of 2020 [28] for the different economic sectors in Aragon and Spain shows that, during COVID-19, there is a smaller impact on the decline in the number of employed that of the Great Recession. In those economic sectors where the sample indicated that ERTE aid was most demanded, the decline in the number of workers employed during the pandemic was smaller than during the Great Recession. The opposite was true in those sectors where ERTEs were less demanded. It appears that ERTE-aid has saved jobs during the COVID pandemic, although our results indicate that savings would have been restricted to the furloughed employees, those who without the aid program would most likely have become unemployed. Now the expectation is that, in the post-COVID period, activity will pick up again and the furloughed employees will soon become active employees once more.

## Supporting information

S1 Appendix (DOCX)

S2 Appendix Sample representativeness, robustness of results, and data quality. (DOCX)

S1 Dataset (XLSX)
