# Supplementary material for: Is there life beyond the Spanish government’s aid to furloughed employees by COVID-19?
Source: PLoS One. 2021 Jun 23;16(6):e0253331. doi: 10.1371/journal.pone.0253331 (PMC8221470; doi:10.1371/journal.pone.0253331)
Supplement: S1 Appendix — (DOCX) [file pone.0253331.s001.docx]

**S1 Appendix**

**S1 Table**. **Credit and macro-financial measures of the Spanish Government against COVID-19**

| **Main Credit and Macro-Financial Measures** |
| --- |
| - It has extended up to €100 billion government guarantees for firms and self-employed, covering both loans and commercial paper of medium-sized companies that participate in Spain’s Alternative Fixed Income Market (MARF); - It launched a new Instituto de Crédito Oficial (ICO) line of guarantees to promote investment activities particularly in the areas of environmental sustainability and digitization (€40 billion); - It created a state rescue fund to support strategic business (€10 billion); - It introduced   - i) €2 billion public guarantees for exporters through the Spanish Export Insurance Credit Company,   - ii) guarantees for loan maturity extensions to farmers using the special 2017 drought credit lines,   - iii) a line of guarantees to provide financial assistance on housing expenses for vulnerable households (€1.2 billion),   - iv) additional loan guarantees for SMEs and self-employed through the Compañía Española de Reafianzamiento (€1 billion). - Additional funding for the ICO credit lines (€10 billion); - Expansion of ICO credit lines for the tourism sector (€200 million); - Loans for the industrial sector to promote digital transformation and modernization (€123.5 million); - Temporary authorization of ICO to participate as a buyer of new commercial paper issued at MARF; - Three-month moratorium on mortgage payments for the most vulnerable, including households, self-employed and homeowners who have rented out their mortgaged properties; - Moratorium on rent payments for vulnerable tenants whose landlord is a large public or private housing holder; - Moratorium on non-mortgage loans and credits, including consumer credits, for the most vulnerable; - Suspension of interest and repayment of loans granted by the Secretariat of State for Tourism for one year with no need for prior request; - Deferred repayment of loans granted to businesses by the Ministry of Industry, Trade, and Tourism; enhanced capacity of the mutual guarantee societies of the autonomous communities; - Deferral of payments on certain loans granted by the Institute for the Diversification and Saving of Energy (IDAE); - Adoption of a mechanism for renegotiation and deferment of business premises rent; - Reduced notary fees for novation of non-mortgage loans; - Ban of short-selling Spanish shares in the stock market from March 16-May 18; - Authorization for special government screening of FDI in strategic sectors; - Adoption of a new macroprudential liquidity tool that   - i) Empowering the National Securities Market Commission to modify requirements applicable to management companies of Collective Investment Schemes;   - ii) Empowering the Consorcio de Compensación de Seguros to act as a reinsurer of credit insurance risks;   - iii) Time-bound changes to corporate resolution frameworks in order to reduce insolvency cases.   - iv) Furthermore, the Bank of Spain will apply to the banks it supervises the flexibility provided by the legal system in relation to the setting of transition periods and the intermediate minimum requirements for own funds and eligible liabilities (MREL) targets; and banks will be allowed to apply expert judgement for the credit-risk classification of forborne exposures. |

Source: [1] and own elaboration
