# Supplementary material for: Is there life beyond the Spanish government’s aid to furloughed employees by COVID-19?
Source: PLoS One. 2021 Jun 23;16(6):e0253331. doi: 10.1371/journal.pone.0253331 (PMC8221470; doi:10.1371/journal.pone.0253331)
Supplement: S2 Appendix — (DOCX) [file pone.0253331.s002.docx]

**S2 Appendix: Sample representativeness, robustness of results, and data quality**

As noted in the main text of the paper, the Government of Aragon used a somewhat informal means of collecting the firm level data that was needed to assess the perceptions of firms about the severity of the unprecedented and unanticipated pandemic, a few weeks after the declaration of the state of alarm (the official recognition of the COVID-19 pandemic in Spain, in the middle of March 2020). Although the “consistency” of the results presented in the main text suggest that the information provided by the survey in early May 2020 should have been useful for policy decisions by the regional government, there remains the issue of the representativeness of the sample of firms that voluntarily answered the survey, and the related issue of the reliability/quality of the responses given by firms To what extent were the prospects for the evolution of activity in the following months, and the prospects for the evolution of employment good predictors of what really happened in the Spanish economy during the months after April 2020 [1]. This appendix presents complementary information on the issues of the representativeness, and the quality of the survey data.

**Representativeness**

The response rate is the first input to consider. Although, in general, a higher response rate is associated with higher representativeness, it will be neither a necessary nor a sufficient condition. It will always be necessary to assess the possibility of bias from non-respondents, and to determine that all the heterogeneous groups of individuals in the population, firms from different sectors and size classes in this case, are represented in the sample data.

One way to test for possible non-respondent bias is to compare the responses of early responders with those of the later ones. For instance, compare the responses of those firms who respond after the first call, with the responses of the firms that respond only late in the process, or indeed on the last day that the survey is open [2].

To test for the possibility of non-response bias, χ^2^ tests were conducted comparing the distribution of early and late respondents across sectors and across size classes. The null hypothesis that the late respondents randomly self-select across sectors in equal proportions to the early respondents was not rejected (χ^2^ (14) = 11.60). A similar result was obtained when the hypothesis was formulated in terms of random self-selection across the different size classes (χ^2^ (3) = 1.68).

The sector and size class representativeness of the sample respondents were assessed testing the null hypothesis that the differences between the proportion of firms in the sample and the proportion of firms in the population, in each sector and in each size class, were within the sample error. For this purpose, we calculated 95% binomial confidence intervals for the proportions of respondents in each sector in the IAF sample. If the proportion of firms in the population in the respective sector falls outside of the confidence interval, the conclusion will be that the sector is over- or infra-represented in the sample. The same exercise is conducted to examine the representativeness of the firms in the sample across size classes.

From Table S2.1, in 8 of the 15 sectors, the 95% confidence interval calculated from the proportion of firms of the sector in the sample data, includes the percentage of firms of the sector in the population of firms in Aragon. In the other 7 sectors, the proportions of firms in the population falls outside the confidence interval and therefore the firms in the sample would either infra- or over-represent them.

**Table S2.1 Distribution of respondents by sector**

|  |  | **Survey IAF-COVID-19** | | **Aragon Economy** |
| --- | --- | --- | --- | --- |
|  |  | % Firms | [95% conf interval] | % Firms |
| **A** | **Agriculture, Forestry and Fishing** | 3.0 | [1.7 4.9] | 6.2 |
| **C** | **Manufacturing** | 26.4 | [22.7 30.4] | 16.3 |
| **D-E** | **Energy and Water** | **1.9** | [0.9 3.4] | 1.1 |
| **F** | **Construction** | **5.3** | [3.5 7.5] | 6.1 |
| **G** | **Wholesale and Retail Trade; Repair of Motor Vehicles and Motorcycles** | **11.7** | [9.1 14.7] | 14.7 |
| **H** | **Transportation and Storage** | 2.1 | [1.0 3.7] | 5.4 |
| **I** | **Accommodation and Food Service Activities** | **5.5** | [3.7 7.8] | 6.7 |
| **J** | **Information and Communication** | 4.3 | [2.8 6.4] | 1.9 |
| **K** | **Financial and Insurance Activities** | **1.7** | [0.8 3.2] | 1.6 |
| **L** | **Real Estate Activities** | **0.9** | [0.3 2.2] | 0.5 |
| **M** | **Professional, Scientific and Technical Activities** | 7.5 | [5.4 10.1] | 3.8 |
| **O** | **Public Administration and Defense; Compulsory Social Security** | 1.3 | [0.5 2.7] | 6.8 |
| **P** | **Education** | 6.4 | [4.5 8.8] | 4.1 |
| **Q** | **Human Health and Social Work Activities** | **4.2** | [2.6 6.2] | 9.3 |
| **S** | **Other Service Activities** | **17.7** | [14.6 21.3] | 15.6 |
|  | **Total** | **100.0** |  | **100.0** |

With respect to the representativeness across firm size, Table S2.2 shows that the population of micro firms is under-represented in the sample data, while the rest of the size classes are over-represented. This result probably has to do with the fact that the micro firms are under-represented in the surveyed population of 5,000 firms that collaborated with the IAF.

**Table S2.2 Distribution by size classes**

|  | **Survey IAF-COVID-19** | | **Aragon Economy 2019** |
| --- | --- | --- | --- |
|  | % Firms | [95% conf interval] | % Firms |
| **Micro (less than 10 employees)** | 49.1 | [44.7 53.4] | 89.6 |
| **Small (10 to 49 employees)** | 31.3 | [27.4 35.5] | 8.7 |
| **Medium (50 to 249 employees)** | 13.2 | [10.4 16.4] | 1.3 |
| **Large (250 or more employees)** | 6.4 | [4.5 8.8] | 0.4 |
| **Total** | **100.0** |  | **100.0** |

Considering the mixed results from the representativeness test, it is recommended to estimate the empirical models weighting the raw data with the proportion of firms in each sector and size class. Not all agree that weighting is the best way to proceed. For instance, [3] writes: "At a time when most surveys have unequal probabilities of selection either by design or by other practical constraints, the question of whether to weight variables during the analysis takes on added importance. If weighting data were a cost-free option, then always weighting would be a reasonable strategy. But unnecessarily weighting means lower efficiency and lower statistical power. Tests that determine whether weights are required do exist, but they are rarely applied for several reasons. One is the lack of awareness among researchers. Another is the influence of tradition in different fields—some always weight and others never do. An additional reason is that some of these tests are not readily available in software packages. Furthermore, even when these tests are easy to implement, there is little guidance on which of the many tests to choose." In a similar vein, see also [4] and [5]. Following [6], the appendix presents the results of fitting the different econometric models, with and without weights, to assess whether the associated confidence intervals (CIs) overlap, which would indicate that the weighting does not make a difference in the results, or does not overlap, in which case the weighting makes a difference. The CI for the weighted estimates will be calculated correcting for the heteroskedasticity that might be introduced with the weighting. Technically, comparing CIs in this way is analogous to a Hausman-type Test of equal coefficients for two differently specified models.

Tables S2.3 and S2.4 show the results of the probit and ordered probit models of Tables 4 and 5 of the main text, respectively, with the sample observations weighted by the proportions of firms per sector and size in the population, and with no weighting at all. The overlap of the CIs is generalized across the explanatory variables in the two tables, indicating that weighting, or not, makes no difference in the empirical results. To simplify the exposition, the results presented in the main text are those obtained with no weighting.

**Table S2.3 Probit estimation: ERTE-aid**

|  | **Unweighted** | | **Weighted** | |
| --- | --- | --- | --- | --- |
|  | **Coef.** | **[95% conf interval]** | **Coef.** | **[95% conf interval]** |
| **Sector** |  |  |  |  |
| Agriculture, Forestry and Fishing | -0.124 | [-1.449 1.201] | -0.909 | [-2.451 0.634] |
| Manufacturing | 0.552* | [-0.071 1.175] | 0.493 | [-0.178 1.164] |
| Energy and water | 0.579 | [-0.476 1.633] | 1.131** | [0.230 2.033] |
| Construction | 0.340 | [-0.438 1.119] | -0.397 | [-1.317 0.524] |
| Wholesale and Retail Trade; Repair of Motor and Vehicles | 0.988*** | [0.315 1.661] | 0.954*** | [0.271 1.637] |
| Transportation and Storage | 0.446 | [-0.787 1.678] | 0.383 | [-0.733 1.500] |
| Accommodation and Food Service Activities | 0.780* | [-0.015 1.575] | 0.717* | [-0.120 1.554] |
| Information and Communication | 0.208 | [-0.653 1.070] | -0.097 | [-1.030 0.835] |
| Financial and Insurance Activities | - |  | - |  |
| Real Estate Activities | 0.845 | [-0.481 2.171] | 0.741 | [-0.860 2.343] |
| Public Administration and Defense; Compulsory Social Secur. | - |  | - |  |
| Education | 0.780** | [0.019 1.542] | 1.482*** | [0.583 2.381] |
| Human Health and Social Work Activities | 0.388 | [-0.420 1.197] | 0.175 | [-0.860 1.209] |
| Other Service Activities | 0.421 | [-0.220 1.061] | 0.488 | [-0.162 1.139] |
| **Size** |  |  |  |  |
| Small | 0.645*** | [0.313 0.978] | 0.815*** | [0.403 1.227] |
| Medium | 0.846*** | [0.406 1.286] | 0.960*** | [0.469 1.451] |
| Large | 1.273*** | [0.538 2.009] | 1.453*** | [0.369 2.538] |
| **Reopening prospects** |  |  |  |  |
| Less than 2 months | 1.383*** | [1.023 1.744] | 1.365*** | [0.746 1.984] |
| Between 2 and 6 months | 1.210*** | [0.845 1.575] | 0.981*** | [0.405 1.558] |
| More than 6 months | 1.509*** | [1.029 1.988] | 1.410*** | [0.667 2.153] |
| **Revenues reduction prospects** |  |  |  |  |
| Between 25% and 40% | -0.242 | [-0.586 0.102] | -0.314 | [-0.794 0.166] |
| Between 10% and 25% | -0.567*** | [-0.976 -0.158] | -0.447 | [-1.007 0.114] |
| Between 5% and 10% | -1.087*** | [-1.606 -0.567] | -1.114*** | [-1.828 -0.401] |
| Up to 5% | -2.145*** | [-2.958 -1.333] | -2.920*** | [-3.773 -2.067] |
| Constant | -1.317*** | [-1.951 -0.683] | -1.204*** | [-1.955 -0.454]] |
| Observations | 514 |  | 514 |  |
| χ^2^(22) | 247.57*** |  | 119.98*** |  |

Note: *Statistically significant at the .10 level; ** at the .05 level; *** at the .01 level. The reference category for sector: Professional, Scientific and Technical Activities; for size: Micro; for reopening prospects: Not discontinued; for revenues reduction prospects: More than 40%.

**Table S2.4 Ordered probit estimation: Employment reduction for** **firms with ERTE-aid or not**

|  | **No ERTE-aid** | | | | **ERTE-aid** | | | |
| --- | --- | --- | --- | --- | --- | --- | --- | --- |
|  | Unweighted | | Weighted | | Unweighted | | Weighted | |
|  | Coef. | [95% conf interval] | Coef. | [95% conf interval] | Coef. | [95% conf interval] | Coef. | [95% conf interval] |
| **Sector** |  |  |  |  |  |  |  |  |
| Agriculture, Forestry and Fishing | -0.590 | [-1.443 0.262] | -0.715 | [-1.796 0.366] | -0.521 | [-2.728 1.686] | -0.939 | [-2.372 0.494] |
| Manufacturing | -0.207 | [-0.769 0.355] | 0.022 | [-0.726 0.770] | -1.052** | [-1.965 -0.139] | -1.410** | [-2.756 -0.064] |
| Energy and water | -0.105 | [-1.196 0.985] | 0.594 | [-0.335 1.524] | -0.677 | [-2.051 0.696] | -0.874 | [-2.306 0.559] |
| Construction | -0.642* | [-1.360 0.077] | -0.481 | [-1.470 0.508] | -1.288** | [-2.388 -0.188] | -1.693** | [-2.999 -0.386] |
| Wholesale and Retail Trade; Repair of Motor and Vehicles | -0.350 | [-1.023 0.324] | -0.036 | [-0.977 0.905] | -0.583 | [-1.515 0.349] | -0.883 | [-2.185 0.418] |
| Transportation and Storage | -0.510 | [-1.585 0.565] | -0.597 | [-1.987 0.793] | -1.097 | [-2.486 0.292] | -2.656*** | [-4.218 -1.094] |
| Accommodation and Food Service Activities | -1.296** | [-2.469 -0.123] | -1.103** | [-2.093 -0.113] | -1.634*** | [-2.629 -0.640] | -2.182*** | [-3.594 -0.770] |
| Information and Communication | -0.416 | [-1.160 0.328] | -0.624 | [-1.666 0.418] | -0.768 | [-1.951 0.416] | -0.889 | [-2.496 0.718] |
| Financial and Insurance Activities | -0.034 | [-1.120 1.052] | -0.547 | [-1.714 0.620] | - |  | - |  |
| Real Estate Activities | -0.386 | [-2.057 1.286] | -0.893* | [-1.816 0.030] | -0.399 | [-2.080 1.282] | -0.984 | [-2.560 0.593] |
| Public Administration and Defense; Compulsory Social Secur. | 0.460 | [-0.699 1.619] | 0.222 | [-0.714 1.157] | - |  | - |  |
| Education | -0.396 | [-1.360 0.077] | -1.454*** | [-2.478 -0.429] | -1.136** | [-2.120 -0.153] | -1.526** | [-2.886 -0.166] |
| Human Health and Social Work Activities | -0.431 | [-1.261 0.400] | -0.626 | [-1.740 0.487] | -1.511*** | [-2.633 -0.390] | -2.066*** | [-3.594 -0.538] |
| Other Service Activities | -0.326 | [-0.895 0.242] | -0.396 | [-1.143 0.351] | -0.969** | [-1.894 -0.043] | -1.283* | [-2.590 0.025] |
| **Size** |  |  |  |  |  |  |  |  |
| Small | -0.210 | [-0.566 0.145] | -0.455** | [-0.882 -0.028] | 0.006 | [-0.355 0.367] | 0.132 | [-0.278 0.542] |
| Medium | -0.344 | [-0.847 0.159] | -0.579** | [-1.103 -0.055] | -0.224 | [-0.678 0.229] | 0.003 | [-0.568 0.574] |
| Large | -0.676** | [-1.323 -0.029] | -0.974** | [-1.837 -0.111] | -0.202 | [-0.896 0.492] | 0.139 | [-0.560 0.837] |
| **Reopening prospects** |  |  |  |  |  |  |  |  |
| Less than 2 months | -0.209 | [-0.646 0.228] | -0.019 | [-0.663 0.625] | -0.015 | [-0.476 0.446] | -0.055 | [-0.899 0.790] |
| Between 2 and 6 months | -0.411* | [-0.850 0.029] | -0.506 | [-1.161 0.150] | 0.026 | [-0.440 0.493] | 0.136 | [-0.751 1.023] |
| More than 6 months | -1.397*** | [-2.123 -0.671] | -2.138*** | [-3.055 -1.221] | -0.123 | [-0.642 0.396] | 0.053 | [-0.860 0.966] |
| **Revenues reduction prospects** |  |  |  |  |  |  |  |  |
| Between 25% and 40% | 0.400* | [-0.054 0.854] | 0.644* | [-0.042 1.330] | 0.647*** | [0.287 1.008] | 0.495** | [0.016 0.974] |
| Between 10% and 25% | 1.153*** | [0.666 1.639] | 1.509*** | [0.806 2.211] | 1.076*** | [0.602 1.550] | 1.089*** | [0.533 1.644] |
| Between 5% and 10% | 1.241*** | [0.701 1.781] | 1.812*** | [1.045 2.580] | 1.617*** | [0.927 2.307] | 1.481*** | [0.794 2.169] |
| Up to 5% | 2.219*** | [1.613 2.825] | 3.170*** | [2.355 3.985] | 1.325* | [-0.195 2.844] | 1.056** | [0.086 2.026] |
| Observations | 292 |  | 292 |  | 238 |  | 238 |  |
| χ^2^(24/22) | 135.37*** |  | 226.43*** |  | 68.15*** |  | 65.71*** |  |

Note: *Statistically significant at the .10 level; ** at the .05 level; *** at the .01 level. The reference category for sector: Professional, Scientific and Technical Activities; for size: Micro; for reopening prospects: Not discontinued; for revenues reduction prospects: More than 40%

**Quality of the survey data**

The assessment of the “quality” of the data collected with the survey will be done comparing the predictions for the future evolution of revenues from sales and of employment, implicit in the prospects for the evolution of these variables that firms in the survey responded to in April-May 2020, with what really happened in the following months. There are two sources: the data reported by the Bank of Spain from the responses of 4,004 Spanish firms (out of 12,494 firms to which the survey was sent) to a survey open for voluntary participation from November 4 to November 19 2020; and the sector data on the actual evolution of employment across economic sectors in Spain during the year 2020, with respect to the level of employment in the fourth quarter of 2019, from the official statistics.

The raw data for the respective comparisons are summarized in Table S2.5 and S2.6. All the numbers in the Table are percentage values. The first two columns compare across sectors –Table S2.5– and across size –Table S2.6– classes the aggregate responses of the firms from the IAF-COVID-19 survey and the responses of the firms from the Bank of Spain survey, EBAE, in terms of prospects for the evolution of revenues. The second block of columns, from 3 to 8, contains information on the evolution of employment from the IAF-COVID-19 survey, the EBAE survey, and from the INE (Spanish Statistical Office) official statistics on the actual evolution of employment up to the fourth quarter of 2020 with respect the fourth quarter of 2019 (only sector data are available). It is necessary to take into account that the INE data includes all employees in the private and public sectors, self-employed and salaried employees, while the EBAE and the IAF survey data contains data only on salaried employees. In addition, the INE data is not homogenized in terms of equivalent full-time employees. This explains the difference in average rates of change in employment calculated with the survey data, -8.6% in the two surveys, and with the actual data, -3.1% from the fourth quarter of 2019 to the fourth quarter of 2020.

**Table S2.5 Revenues and employment prospects by economic sectors, from IAF and Bank of Spain surveys. Comparison with 2020 official employment data.**

|  |  | **Prospects for the evolution of revenues (%)** | | **Prospects and actual data for the evolution of** **employment (%)** | | | | | | |
| --- | --- | --- | --- | --- | --- | --- | --- | --- | --- | --- |
|  |  | Survey IAF-COVID-19 | EBAE^a^ | Survey IAF-COVID-19 | EBAE^a^ | INE^b^ | | | |  |
|  |  | R-IAF | R-EBAE | E-IAF | E-EBAE | E-T4 | E-T3 | E-T2 | E-T1 |  |
| **A** | **Agriculture, Forestry and Fishing** | -8.9 |  | -3.0 |  | -1.5 | -7.9 | -3.8 | -1.1 |  |
| **C** | **Manufacturing** | -25.7 | -12.7 | -7.9 | -5.5 | -2.7 | -3.3 | -4.4 | 0.3 |  |
| **D-E** | **Energy and Water** | -19.3 |  | -3.8 |  | -0.9 | 3.0 | -4.3 | -1.3 |  |
| **F** | **Construction** | -23.5 | -12.2 | -8.3 | -5.2 | -0.3 | -2.7 | -8.9 | -0.5 |  |
| **G** | **Wholesale and Retail Trade; Repair of Motor Vehicles and Motorcycles** | -33.1 | -15.6 | -8.8 | -7.3 | -4.4 | -5.0 | -8.1 | -1.8 |  |
| **H** | **Transportation and Storage** | -24.6 | -16.3 | -8.9 | -8.3 | -3.9 | -6.6 | -8.9 | -3.0 |  |
| **I** | **Accommodation and Food Service Activities** | -44.9 | -45.5 | -19.9 | -35.0 | -23.3 | -12.2 | -19.3 | -6.8 |  |
| **J** | **Information and Communication** | -25.1 | -11.3 | -6.7 | -3.3 | -0.7 | 1.5 | 2.8 | 0.9 |  |
| **K** | **Financial and Insurance Activities** | -14.5 |  | -4.0 |  | 9.5 | 3.0 | -2.2 | -0.9 |  |
| **L** | **Real Estate Activities** | -19.5 | -10.3 | -6.0 | -3.2 | 4.3 | 3.3 | -0.9 | 1.5 |  |
| **M** | **Professional, Scientific and Technical Activities** | -25.3 | -10.0 | -5.2 | -5.0 | 2.2 | -1.6 | -1.6 | 0.2 |  |
| **O** | **Public Administration and Defense; Compulsory Social Security** | -20.7 |  | -3.9 |  | 1.8 | 2.2 | -3.0 | -0.4 |  |
| **P** | **Education** | -28.3 |  | -9.8 |  | 1.3 | -11.7 | -6.1 | 1.2 |  |
| **Q** | **Human Health and Social Work Activities** | -26.5 |  | -11.3 |  | 3.3 | 5.1 | -1.1 | -2.0 |  |
| **S** | **Other Service Activities** | -29.0 | -27.6 | -9.1 | -17.2 | -5.8 | -5.2 | -8.7 | -2.8 |  |
|  | **Total** | **-27.2** | **-16.1** | **-8.6** | **-8.6** | **-3.1** | **-4.0** | **-6.8** | **-1.4** |  |

Note: ^a^ EBAE: Bank of Spain Business Activity Survey

^b^ INE (Spanish Statistical Office), 2021: Employment reduction based on the fourth quarter of 2019 for the four quarters of 2020.

R- denotes revenues. E- denotes employment.

**Table S2.6 Revenues and employment prospects by firm size, from IAF and Bank of Spain surveys. Comparison with 2020 official employment data.**

|  | **Prospects for the evolution of revenues (%)** | | **Prospects and actual data for the evolution of employment (%)** | |
| --- | --- | --- | --- | --- |
|  | Survey IAF-COVID-19 | EBAE^a^ | Survey IAF-COVID-19 | EBAE^a^ |
|  | R-IAF | R-EBAE | E-IAF | E-EBAE |
| **Micro (less than 10 employees)** | -31.2 | -19.0 | -9.8 | -10.4 |
| **Small (10 to 49 employees)** | -24.0 | -17.9 | -6.9 | -10.0 |
| **Medium (50 to 249 employees)** | -25.1 | -12.6 | -8.5 | -7.2 |
| **Large (250 or more employees)** | -16.8 | -12.4 | -6.6 | -5.9 |
| **Total** | **-27.2** | **-16.1** | **-8.6** | **-8.6** |

Note: ^a^ EBAE: Bank of Spain Business Activity Survey.

R- denotes revenues. E- denotes employment.

Table S2.5 is complemented with Table S2.7 showing the Pearson’s simple pair-wise correlations among the variables. Overall, the data from the IAF COVI-19 survey correlates highly with the data of the other survey and with the actual data. This correlation supports two assertions. First, that what happened in Aragon’s business sector is quite representative of what happened at the country level. And second that the aggregation of the firm level information collected in April-May 2020, one year later, has proven to be a good predictor of the rest of the year evolution of the sectors actual data aggregates on business activity and employment.

**Table S2.7 Pearson correlations matrix among the variables from the data in Table S2.5 and S2.6**

|  | **R-IAF** | **R-EBAE** | **E-IAF** | **E-EBAE** | **E-T4** | **E-T3** | **E-T2** | **E-T1** |
| --- | --- | --- | --- | --- | --- | --- | --- | --- |
| **R-IAF** | 1.000 |  |  |  |  |  |  |  |
| **R-EBAE** | 0.797 | 1.000 |  |  |  |  |  |  |
| **E-IAF** | 0.893 | 0.926 | 1.000 |  |  |  |  |  |
| **E-EBAE** | 0.806 | 0.996 | 0.932 | 1.000 |  |  |  |  |
| **E-T4** | 0.756 | 0.950 | 0.782 | 0.949 | 1.000 |  |  |  |
| **E-T3** | 0.464 | 0.709 | 0.529 | 0.710 | 0.666 | 1.000 |  |  |
| **E-T2** | 0.668 | 0.850 | 0.746 | 0.854 | 0.838 | 0.727 | 1.000 |  |
| **E-T1** | 0.570 | 0.907 | 0.694 | 0.904 | 0.788 | 0.419 | 0.807 | 1.000 |

Note: R- denotes revenues. E- denotes employment.

**References**

1. Fan W, Yan Z. Factors affecting response rates of the web survey: A systematic review. Comput Hum Behav. 2010; 26, 132-139.
2. Yessis J, Rathert C. Initial versus Prompted Responders to Patient Satisfaction Surveys: Implications for Interpretation and Patient Feedback. J Appl Manag Entrep. 2006, 11, 49-64.
3. Bollen K, Biemer P, Karr A, Tueller S, Berzofsky M. Are Survey Weights Needed? A Review of Diagnostic Tests in Regression Analysis. Annu Rev Stat Appl. 2016, 3, 375-392.
4. Solon G, Haider S, Wooldridge J. What Are We Weighting For? J Hum Resour. 2015, 50, 301-316
5. Gelman A. Struggles with Survey Weighting and Regression Modelling. Statist Sci. 2007, 22, 153-164.
6. Hahs-Vaughn D, Lomax R. Utilization of sample weights in single-level structural equation modelling. J Exp Educ. 2006, 74, 161-190
